# The enclosed ward management strategies in psychiatric hospitals during COVID-19 outbreak

**DOI:** 10.1186/s12992-020-00586-z

**Published:** 2020-06-24

**Authors:** Jiajia Chen, Maoxiang Xiong, Zongling He, Wen Shi, Yuchuan Yue, Manxi He

**Affiliations:** 1 The Clinical Hospital of Chengdu Brain Science Institute, School of Life Science and Technology, University of Electronic Science and Technology of China, Chengdu, Sichuan China; 2 The Mental Health Centre of Chengdu, Sichuan, 610036 China

**Keywords:** Psychiatric hospital, COVID-19 pandemic, Enclosed wards, Management strategies

## Abstract

During the COVID-19 pandemic, as a large city located in Southwest China, Chengdu was mainly affected by imported cases. For a psychiatric hospital, the enclosed management model, the crowded wards and the uncooperative patients are the risk factors of nosocomial infection. Admitting new patients while preventing the COVID-19 outbreak within the institutions was a crucial challenge. The Mental Health Centre of Chengdu proposed a series of effective management strategies to deal with the rapidly evolving situation during the COVID-19 pandemic which included regulation for the inpatients, their families and staff, and achieved Zero infection in our hospital.

In December 2019, novel coronavirus pneumonia (also called COVID-19) emerged in Wuhan and soon spread to other large cities in China. In early February 2020, due to nosocomial infection, 80 patients and staff from the Wuhan Mental Health Centre were diagnosed with COVID-19 [1], whereas 119 people were confirmed to be infected in Daenam Hospital, South Korea, in early March [2]. For psychiatric hospitals, the enclosed management model, crowded wards and uncooperative patients are risk factors for hospital-associated infection [3]. Admitting new mental health patients while preventing hospital infection was a crucial challenge. The largest mental health center in southwestern China, the Mental Health Centre of Chengdu, adopted a series of effective management strategies to address the rapidly evolving situation and successfully achieved the goal of zero infection. Our 5 stages of coping strategies followed the timeline of the pandemic, as described below.

## Stage one

At the beginning of the outbreak (mid-January 2020), we immediately formed a COVID-19 prevention and control leadership group and established enclosed ward management regulations, such as temporarily stopping inpatient admission and prohibiting visits (families contacted patients via video call instead) [4]. In each ward, we set up two observation rooms for inpatients, in case they had fever or other respiratory symptoms. Specific regulations were formulated for patients’ relatives, caregivers and janitors, requiring them to stay in the wards 24 h/day and 7 days/week. All the food and drinks were served by the canteen, where the staff’s health condition was carefully monitored. No take-away food was allowed.

## Stage two

At the end of January 2020, the COVID-19 epidemic was expanding all over China. The Chengdu local government required all the public hospitals to help treat diagnosed cases and suspected cases. Our hospital was a designated hospital for suspected cases, which increased our risk of COVID-19 exposure. We promptly set up an isolation ward for suspected cases on the top floor of the inpatient building, which was a former clinical trial lab with an individual sewer system and air-purifying system, and assembled the top doctors of our hospital to form the medical team. To avoid cross infection, we optimized a special route to the isolation ward and disabled all the air-conditioners. All staff and their families had to follow specific regulations, which required them to report their personal health condition and whereabouts daily. They were also advised to stay home after work, avoid gatherings or leaving Chengdu, and try not to take any public transport -a transport fund was given as a financial support.

## Stage three

In mid-February 2020, the COVID-19 pandemic reached its peak in China. We were at the patient intake peak of the year. To meet the substantial demand for hospitalization, we set up a transitional observation ward with 60 beds. In the observation ward, new patients were observed in separated rooms for 14 days [3] until the relevant standards were met, at which point they were transferred to a regular ward. Staff who worked in this ward could not return home until the patients were confirmed to be noninfectious. If a staff member had close contact with a confirmed case, then they would have to go through a 14-day observation and COVID-19 nucleic acid test.

## Stage four

At the end of February, the pandemic remained at the peak. To avoid cross infection, we placed new patients with and without fever into two separate areas. We mobilized a ward with 40 beds for the suspected patients with fever called the “fever ward”. As a supplement for the observation ward, we enacted total physical isolation for different patients. At that point, our hospital formulated a mature procedure for patient transfer from the fever ward/observation ward to the regular ward.

## Stage five

At the end of March, as the pandemic eased in China, there were no new cases reported in Chengdu for several consecutive days. We disassembled the observation wards while maintaining the whole manpower structure. Instead of observation wards, in each regular ward, we installed two areas: one was a “fever area”, and the other was an observation area. New patients were assigned to these two areas according to their physical condition. COVID-19 nucleic acid examination and chest CT scan were performed among all new patients to eliminate the risk of carrying the novel coronavirus. After 14 days of observation, if certain criteria (negative nucleic test result, negative chest CT result and normal body temperature for 3 days) [5] were met, these new patients were transferred to general rooms.

As the understanding of COVID-19 is constantly updated, we have adjusted our prevention strategies in a timely manner. When the pandemic situation improved, we suggested that staff could leave Chengdu but not Sichuan Province; otherwise, they will have to go through 14-day self-quarantine and COVID-19 nucleic acid tests. In the summer, we will fix separate air-conditioners with air-purifying functions in each ward.

As stated above, psychiatric hospitals are facing critical administrative challenges in this pandemic. We hope that our experiences can serve as a reference for other mental health hospitals in terms of minimizing the adverse outcomes of this pandemic for the mentally ill population.

## Data Availability

Not applicable.
